# Prevalence of decreased olfactory function in adults in a Brazilian population sample

**DOI:** 10.1016/j.bjorl.2025.101695

**Published:** 2025-08-05

**Authors:** Wilker Antunes Lira, Gilmar Felisberto Junior, Marco Aurélio Fornazieri, Wilma Terezinha Anselmo Lima, Ingrid Werner Picinini, Vanessa Ramos Pires Dinarte

**Affiliations:** a Faculdade de Medicina de Marília (FAMEMA), Marília, SP, Brazil; b Universidade Estadual de Londrina (UEL), Departamento de Cirurgia Clínica, Londrina, PR, Brazil; c Universidade de São Paulo (USP), Faculdade de Medicina de Ribeirão Preto, Departamento de Otorrinolaringologia, Ribeirão Preto, SP, Brazil

**Keywords:** Olfaction, Prevalence, Quality of life, Smell, Anosmia

## Abstract

**Objective:**

To investigate the prevalence of undiagnosed olfactory changes in individuals over 40 and the associated factors.

**Methods:**

A cross-sectional study enrolled a group of adult and elderly patients who did not report any nasal issues or olfactory changes with a clear or highly probable causal connection. They were subjected to the University of Pennsylvania Smell Identification Test (UPSIT) and the Visual Analog Scale to evaluate olfactory capacity.

**Results:**

Two hundred eighty-four volunteers participated. Among the elderly, 96.9% experienced olfactory changes, while 80.4% of the younger group did. There was a significant correlation between age and UPSIT results (Rho = 0.33, p < 0.001) in the elderly. A higher prevalence of olfactory impairment was found in the elderly (p < 0.001). The age predictor showed an OR of 1.081 (p < 0.01, 95% CI 1.03–1.12). Both elderly and adults reported similar rates of altered olfaction, at 14.5% and 14.3%, respectively.

**Conclusion:**

There was a higher prevalence and risk of developing decreased olfaction in the elderly, with an 8% greater risk of olfactory dysfunction for each passing year.

**Level of evidence:**

5.

## Highlights

• Older individuals with an 8% increased risk of developing loss of smell.
• Low correlations between self-reported olfactory function and objective performance.
• The sense of smell is often underestimated.

## Introduction

Over two hundred causes can be related to changes in olfaction, such as rhinosinusitis. Moreover, the olfactory epithelium undergoes physiological degeneration as time passes. However, the elderly are susceptible to the same causes that provoke Olfactory Dysfunction (OD) in all other age groups. Therefore, it is often difficult to distinguish between chemosensory changes resulting from the normal aging process, diseases, and the side effects of medications they use.^1^

Despite Pinto et al.^2^ showing that olfactory complaints led to over 200,000 medical consultations annually in the United States (US), olfaction has received less attention than other senses. However, the COVID-19 pandemic emphasized the crucial importance of this sense in human life.

From this perspective, several epidemiological studies in other countries have explored the prevalence of OD, demonstrating rates ranging from 1.4% to 62.4%,^3^ which can reach 75% among older adults.^4^ This variation likely reflects differences in testing methods, criteria for defining dysfunction, sample sizes, sampling procedures, and variations in sex, age, health, and ethnic composition of these populations.^5^

In this context, it is crucial to note that no published articles showed a higher prevalence of olfactory dysfunction in the elderly in Brazil and Latin America through comparisons between younger and older age groups. Thus, the aim of this study was to assess the incidence of reduced olfaction in a specific region of Brazil and to establish its correlation with age.

## Methods

This cross-sectional study was conducted at the Clinics Hospital of Marília Medical School (HCFAMEMA), São Paulo, Brazil. It was conducted between July 2022 and March 2023. The Research Ethics Committee of Marília Medical School reviewed and approved the project under protocol number 59523722.5.0000.5413.

A group of 284 participants was enrolled and split into two categories: elderly (60 years or older), consisting of 131 individuals, and younger adults (40–59 years), comprising 153 individuals. Individuals aged 40 and above who were waiting for care in different outpatient clinics at HCFAMEMA and did not have nasal issues as their main reason for seeking consultation were randomly enrolled in the study. This way if someone

To investigate the connection between olfactory loss and aging, the study did not include individuals with a potential olfactory change that was clearly or highly likely to have a specific cause. This included those with nasal complaints as their main reason for consultation, current antibiotic use, acute or chronic rhinosinusitis, current or recent upper respiratory tract infection, or possible causal factor of olfactory loss (like COVID-19), previous nasal or neurological surgery, history of head trauma as a potential cause of olfactory loss, psychiatric disorders (except anxiety and depression), neurological diseases (Parkinson's disease, Alzheimer's disease, epilepsy, severe memory problems), or any current clinical condition that could compromise the accuracy of the collected data. Exclusion criteria were established based on patient self-reporting and clinical evaluation conducted by the interviewer.

Patients at HCFAMEMA were randomly selected and asked to join the study after reading and signing the Informed Consent Form (ICF). After being accepted, a medical history review was conducted to confirm their eligibility based on the inclusion criteria and to check for any exclusion criteria. Following this, they finished the questionnaire, which was entirely filled out by the examiner.

All patients underwent the University of Pennsylvania Smell Identification Test (UPSIT, Portuguese version, Sensonics International, NJ, USA) to assess olfaction. Based on the number of correct responses, patients were classified individually as having normosmia, mild, moderate, or severe hyposmia, or anosmia, according to the normative table for Brazilians. Patients were only informed of their results at the end of the interview and everyone with positive findings were invited to being included at otorhinolaryngology service to identifying the cause and starting the treatment for their loosing of the smell.

All participants underwent the Visual Analog Scale (VAS) to assess the influence of olfaction on quality of life. During the VAS self-assessment, the scale was presented on an electronic device. Participants were asked to rate their sense of smell on a scale of 0–100 mm, with 0 being no impact and 100 mm being the most significant possible impact, in five situations: how bothersome are odor difficulties to you; how often do you become aware of/concerned about your olfactory disorder; how severely has your olfactory problem affected your professional performance in the last month; how severely has your olfactory disorder affected your recreational activities in the past month; how severely has your olfactory disorder affected your private life in the past month.

### Statistical analysis

Using a Spearman correlation matrix, we analyzed correlations between olfactory test results and ordinal and quantitative qualitative variables for each group. The estimate of olfactory disorder in relation to age, socioeconomic, smoking and others qualitative and quantitative factors shown was evaluated through a simple logistic regression model with Nagelkerke's measure of adjustment. In this analysis, olfactory disorder was treated as a dichotomous variable. The Mann-Whitney *U* test was used to compare olfactory changes between elderly and young groups. All analyses were considered significant when p-values were less than 0.05. Statistical analysis was conducted using Jamovi software, version 2.3.28.

## Results

The elderly patient group consisted of 131 individuals, with an average age of 69.01 ± 6.8 years. The young patient group included 153 patients, averaging 49.82 ± 5.6 years (Table 1). It is noteworthy that HCFAMEMA serves a region with an approximate population of 1,200,000. Therefore, this study shown that people suffering from some olfactory dysfunctions are 2,08 every 10.000 inhabitants.

**Table 1:** Demographic data of included patients.

	Elderly		Younger	
Middle Age	69.01 (59–87)		49.82 (36–59)	
Sex	Male	Female	Male	Female
	48 (36.6%)	83 (63.4%)	39 (25.5%)	114 (74.5%)
Ethnicity	White	94 (72.3%)	White	102 (66.7%)
	Afrodescendant	21 (16.2%)	Afrodescendant	22 (14.4%)
	Mixed Race	12 (9.2%)	Mixed Race	26 (17%)
	Asian	2 (1.5%)	Asian	3 (2%)
	Native	1 (0.8%)	Native	0
Income^a^	Less than 1	6 (4.7%)	Less than 1	11 (7.2%)
	Between 1 and 4	118 (91.5%)	Between 1 and 4	124 (81%)
	Between 5 and 10	5 (3.9%)	Between 5 and 10	16 (10.5)
	More than 10	0	More than 10	6 (3.9%)
Education Level	Incomplete Elementary	64 (49.2%)	Incomplete Elementary	28 (18.3%)
	Complete Elementary	21 (16.2%)	Complete Elementary	22 (14.4%)
	Incomplete Middle	8 (6.2%)	Incomplete Middle	6 (3.9%)
	Complete Middle	27 (20.8%)	Complete Middle	61 (39.9%)
	Incomplete Higher	2 (1.5%)	Incomplete Higher	4 (2.6%)
	Complete Higher	8 (6.2%)	Complete Higher	32 (20.9%)

a Minimum wages.

### Subjective classification of olfaction regardless of UPSIT results

A subjective classification of altered olfaction was reported by 14.5% of elderly participants and 14.3% of young subjects. The correlation analysis between UPSIT results and subjective olfaction classification did not show significance in either group.

### Olfactory test results in each group

**Fig. 1:**
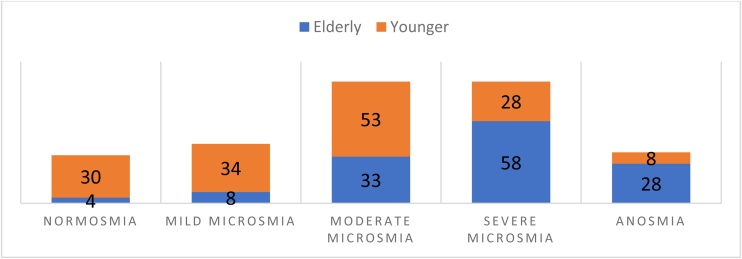
Results of olfactory capacity according to the University of Pennsylvania Smell Identification Test (UPSIT) in adults and elderly. Source: Compiled by the Authors.

In the elderly patients, 127 (96.9%) presented altered olfaction, with 58 (44.3%) classified as having severe hyposmia (Fig. 1). The correlation analysis between age and UPSIT results showed significance (Rho = 0.33 – p < 0.001).

In the young patient group, 123 (80.4%) presented altered olfaction, with the majority (34.6%) showing moderate hyposmia (Fig. 1). The correlation analysis between age and UPSIT results did not show significance in this group (Rho = 0.013 – p = 0.87).

The younger patient group had a lower prevalence of olfactory disorder (p < 0.001). The simple logistic regression also showed a difference between the groups (p < 0.001), and for the age predictor, the regression model showed an OR of 1.081 (p < 0.01) with a 95% CI of 1.03 to 1.12.

### Characteristics of patients with altered UPSIT results

Among the elderly population, there was a greater occurrence of olfactory disorder in female patients (62.9%), white individuals (72.2%), those earning one to four minimum wages (91.3%), and those with incomplete elementary education (50.8%). In the young group, the prevalence was higher among female patients (79.6%), white individuals (67.4%), those with an income of one to four minimum wages (84.5%), and those with complete high school education (38.2%).

Regarding BMI, in both groups, there was a higher prevalence of patients who were overweight, with 48 patients (37.7%) in the elderly group and 47 (38.2%) in the young group.

### Lifestyle and medical background of the studied population

The elderly group had a higher number of individuals using dental prostheses than the young group, with 37 (24.2%) in the young group and 85 (64.9%) in the elderly group. Both groups had similar practices of nasal irrigation. However, the young group had slightly higher usage of corticosteroids, antibiotics within the past month, nasal decongestants, and reported higher rates of current and past smoking and alcohol consumption.

**Fig. 2:**
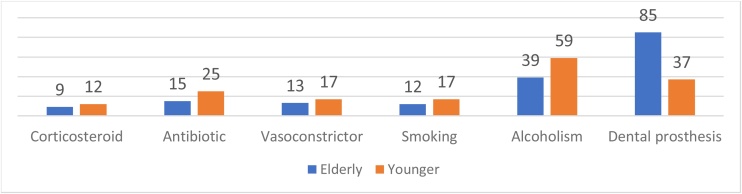
Lifestyle habits of the studied population. Source: Compiled by the Authors.

In Fig. 2, an analysis was conducted to determine if there was a correlation between UPSIT results and smoking, alcohol consumption, and the use of dental prostheses. However, no positive correlation was identified in either group.

There were reports of previous COVID-19 infection in 52 (39.7%) patients in the elderly group and 87 (56.8%) in the young group. However, no correlation was observed between UPSIT results and previous COVID-19 infection in either group.

### Impact on quality of life

In the elderly group, 111 (87.4%) patients reported no impact of olfaction on their quality of life. Among the young group, 113 (91.8%) did not observe that their olfactory capacity affected their well-being.

**Fig. 3:**
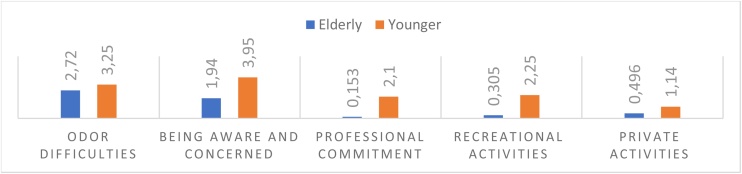
Average scores for each question in the VAS. Source: Compiled by the Authors.

The correlation analysis between the VAS results and the UPSIT did not show significance for the elderly group regarding the scores given. In the young group, there was a correlation between UPSIT results and awareness and concern (Rho = 0.299 – p < 0.001), professional impairment (Rho = 0.215 – p = 0.008), recreational activities (Rho = 0.190 – p = 0.019), and private activities (Rho = 0.242 – p = 0.003). Fig. 3 displays the average scores for each question in the two groups.

## Discussion

This is the first study in Latin America to investigate the prevalence of olfactory alteration, emphasizing the greater presence of olfactory dysfunction in the aging process. It was noted that older individuals were more likely to experience olfactory dysfunction than younger individuals, with an 8% increased risk of developing loss of smell for each additional year of life. Furthermore, it was evident how the sense of smell is often underestimated, as many patients with psychophysical olfactory reduction did not acknowledge any subjective changes. The assessment of the impact of olfactory dysfunction on their quality of life using the VAS revealed low scores.

The high prevalence observed in our study, compared to that reported in the literature, can be attributed to all patients undergoing the 40-question UPSIT assessment. It is known that the prevalence of OD tends to be higher when extended identification tests (> 8 items) are used compared to short tests of 8 items, which seem to miss about 10%–15% of the OD that could be detected with more extended tests.^3^ Also, more extended tests are less prone to random measurement errors and, therefore, have greater reliability.^6^

In our sample, as in epidemiological surveys examining the prevalence of olfactory dysfunction in the general population, despite methodological discrepancies, it has been consistently shown that the prevalence of olfactory dysfunction is high and increases with age^3^, ^4^, ^5^^,^^7^^,^^8^ both in terms of detection thresholds and odor identification.^9^^,^^10^

Anatomical, morphological, and histological changes associated with aging affect odor processing in the olfactory and limbic systems. From this perspective, the literature indicates that genetic and demographic factors play a role in controlling this process.^11^ As a result, due to disparities in sex, age, health status, and ethnic makeup within populations, along with differences in study design, participant selection, and methods of olfactory assessment in various studies,^3^^,^^5^ it is not possible to directly compare our results with those from other regions nationally or globally.^5^

Despite recent longitudinal research suggesting that self-reported OD generally reflects objective olfactory decline in cognitively intact adults,^12^ previous reports from cross-sectional studies and our results indicate low correlations between self-reported olfactory function and objective olfactory performance.^13^ Thus, it was observed that the significant difference between the subjective evaluation of the patients in this study and the psychophysical findings from UPSIT aligns with previous data, which have shown that the prevalence of olfactory impairment based on self-assessment is lower than that based on olfactory tests.^7^ Less than one-third of objectively affected individuals were aware of their olfactory loss.^3^

This discrepancy is likely due to the common unawareness of olfactory deterioration, especially in the elderly.^7^ Subjective evaluations depend on an individual's ability to assess their olfactory function, while psychophysical tests inherently remove the influence of individual perception bias from the evaluation results. Thus, our data reinforce previous findings that self-assessment fails to identify a sizable portion of individuals who do not recognize their olfactory deficits, making it an insensitive measure for detecting OD.^3^^,^^14^

These findings are concerning, as it has been shown that, in relation to Parkinson's Disease (PD), unawareness of olfactory decline has a more significant influence on cognitive decline and the conversion to dementia than being aware of it.^15^ Moreover, it has been suggested that rapid olfactory decline during normal cognition, using repeated olfactory measurement, predicted subsequent cognitive impairment and dementia, highlighting its potential as a simple biomarker for early detection of Alzheimer's Disease (AD).^16^ Therefore, olfactory testing, especially in the elderly, is essential.

Indeed, we found a higher prevalence of OD in females, but it is possible to find mixed results among publications. While some studies indicate comparable data or lack of statistical disparities in the occurrence of olfactory dysfunction between men and women,^8^^,^^9^^,^^17^, ^18^, ^19^ most literature suggests a greater prevalence of olfactory impairment in men.^5^^,^^7^^,^^20^, ^21^, ^22^, ^23^ Whether there are sex- or gender-related distinctions in olfactory ability remains a topic of ongoing research, with varying and conflicting empirical findings and theoretical interpretations.^24^

While methodological differences may contribute to inconsistent associations between olfactory impairment and some factors, another potential, though little-studied, influence is the effect of race or ethnic-regional group.^25^ Xu et al.^10^ did not observe differences in the presence of OD by race, but other publications believe that ethnicity appears to influence olfactory decline. In contrast to our findings in the sample, published research indicates that the prevalence of anosmia is greater among Black individuals than white individuals in the elderly population.^5^^,^^7^^,^^8^

The impact of income and education on olfactory dysfunction is uncertain and conflicting. Based on the hypothesis that individuals with less education and/or income might not be familiar with certain types of odors and therefore score lower, underestimating their “true” odor identification abilities due to their limited exposure or knowledge of such stimuli – since odor identification involves high-level cognitive processes –^8^^,^^26^ it is possible to justify the findings of some cross-sectional studies suggesting that those with low family income and educational level are more likely to suffer from olfactory dysfunction.^5^^,^^8^^,^^17^ However, longitudinal studies do not reinforce the impact of educational and financial status on the rates of cognitive and olfactory decline related to age.^18^^,^^20^^,^^22^

Additionally, it has been shown that people suffering from olfactory dysfunction complain of dissatisfaction and/or food disturbances, which might explain why individuals exhibit poor eating habits and consequently risk developing malnutrition and/or weight gain.^27^ Furthermore, it has been observed that olfactory performance regarding identification, discrimination, and sensitivity to odors is low in individuals with high BMI.^28^ Therefore, the decreased olfactory function found in this study, more commonly in overweight patients, might be caused by hormonal and metabolic changes associated with obesity, such as the negative effect of high insulin resistance on food odor sensitivity.^28^

Factors like recent use of corticosteroids, antibiotics, and nasal decongestants, smoking, alcohol consumption, and the use of dental prostheses, which may affect a patient's sense of smell, were not found to be significant. This suggests that aging may play a more substantial role in this process.

It is important to highlight that, even in the context where the data collection occurred during/post-COVID-19 pandemic, we did not observe statistical significance in both groups. Similarly, to the study by Lechien, Vaira, and Saussez,^27^ older patients had COVID-19-related olfactory dysfunction less frequently than younger patients. As a result, the older patients in our study exhibited more severe olfactory loss and broader disturbances in olfactory function compared to the younger patients. This suggests that the age-related findings may indicate underlying baseline differences in olfactory function between young and elderly individuals rather than variations in the recovery process following infection.^27^

Given that most patients did not mention a loss of smell or were not conscious of their reduced sense of smell, it was anticipated that the ratings on the VAS regarding the effect of the diminished ability to perceive odors on their quality of life would be low. Considering this outcome, it is essential to inform the population, particularly the elderly, about the risks and evidence associated with unintentional or undervalued OD experiences. For example, it has been observed that olfactory deficiency is considered a solid and independent risk factor for death, regardless of the individual's health status.^6^^,^^14^, ^29^, ^30^

Despite the data presented above and the global literature demonstrating that olfactory alterations are influenced by a series of demographic and health factors, the exact relationship between them remains not fully understood, and the extent to which they contribute to this association remains an unanswered question. Further investigation into the underlying mechanisms of these observed associations will be crucial for improving understanding of the link between OD and its causes and consequences.

It is important to mention that olfactory disorders are well-studied in the adult population, but there is a paucity of literature characterizing olfactory dysfunction in the pediatric population. It is assumed that OD in children is relatively uncommon; thus, approximately two percent of patients in smell and taste outpatient clinics are children up to 18 years old. Smell loss at a young age can be indicative of some genetic disorders, but the etiology of OD is due to several underlying mechanisms and causes: post-infectious, sinonasal disease, post-traumatic, iatrogenic, and idiopathic. Congenital and post-traumatic reasons of OD are more frequent at an early age. It changes with increasing age: congenital and post-traumatic cases are replaced by post-infectious and idiopathic OD.^31^^,^^32^

However, it is necessary to emphasize the limitations of this study. Due to Brazil's significant territorial, cultural, and climatic diversity, it is not feasible to generalize the data collected from a single region to reflect the prevalence of OD nationwide accurately. Therefore, gathering data from various regions is essential to conduct a multicentric analysis. Moreover, the occurrence of OD in the general population might be considerably higher as individuals who were at an elevated risk of developing OD were not included in the study.

Since this is a cross-sectional study, we can only establish associations between variables, which hinders the ability to determine the temporal sequence and draw conclusions about causal relationships between variables and olfactory changes. Furthermore, we evaluated only one aspect of olfactory function: odor identification. Due to the lack of resources and the time required to perform the test, chronic condition status, cognitive status, and other variables were evaluated based on self-reported data and the subjective clinical evaluation of the interviewer.

## Conclusion

It is possible to suggest that a sizable portion of Brazilians experience olfactory disorders, indicating a higher occurrence of olfactory dysfunction among elderly individuals. Relying solely on self-assessment of one's sense of smell may not be enough to identify any impairment accurately. Therefore, psychophysical assessments should be conducted on a more regular basis. Despite the COVID-19 pandemic highlighting the importance of olfaction, this sense appears to remain undervalued, as patients with significant deficits did not perceive the alteration and/or did not observe an impact on their quality of life.

## ORCID ID

Wilma Terezinha Anselmo Lima: 0000-0001-9146-2320

Ingrid Werner Picinini: 0009-0003-7051-7822

## Funding

This research did not receive any specific grant from funding agencies in the public, commercial, or not-for-profit sectors.

The project was reviewed and approved by the Research Ethics Committee of Marília Medical School under protocol number 59523722.5.0000.5413.

Consent was obtained from patients to participate in this study and for its publication through written and verbal means.

## Declaration of competing interest

The authors declare no conflicts of interest.
